# Lifestyle Intervention With Smartphone App and isCGM for People at High Risk of Type 2 Diabetes: Randomized Trial

**DOI:** 10.1210/clinem/dgad639

**Published:** 2023-11-01

**Authors:** Masaru Kitazawa, Yasunaga Takeda, Mariko Hatta, Chika Horikawa, Takaaki Sato, Taeko Osawa, Masahiro Ishizawa, Hiroshi Suzuki, Yasuhiro Matsubayashi, Kazuya Fujihara, Takaho Yamada, Hirohito Sone

**Affiliations:** Department of Hematology, Endocrinology, and Metabolism, Niigata University Faculty of Medicine, Niigata 951-8510, Japan; Department of Hematology, Endocrinology, and Metabolism, Niigata University Faculty of Medicine, Niigata 951-8510, Japan; Department of Hematology, Endocrinology, and Metabolism, Niigata University Faculty of Medicine, Niigata 951-8510, Japan; Department of Health and Nutrition, University of Niigata Prefecture Faculty of Human Life Studies, Niigata 950-0806, Japan; Department of Hematology, Endocrinology, and Metabolism, Niigata University Faculty of Medicine, Niigata 951-8510, Japan; Department of Hematology, Endocrinology, and Metabolism, Niigata University Faculty of Medicine, Niigata 951-8510, Japan; Department of Hematology, Endocrinology, and Metabolism, Niigata University Faculty of Medicine, Niigata 951-8510, Japan; Department of Hematology, Endocrinology, and Metabolism, Niigata University Faculty of Medicine, Niigata 951-8510, Japan; Department of Hematology, Endocrinology, and Metabolism, Niigata University Faculty of Medicine, Niigata 951-8510, Japan; Department of Hematology, Endocrinology, and Metabolism, Niigata University Faculty of Medicine, Niigata 951-8510, Japan; Department of Hematology, Endocrinology, and Metabolism, Niigata University Faculty of Medicine, Niigata 951-8510, Japan; Department of Hematology, Endocrinology, and Metabolism, Niigata University Faculty of Medicine, Niigata 951-8510, Japan

**Keywords:** continuous blood glucose monitor, prevention, prospective randomized trial, smartphone, educational intervention

## Abstract

**Aims:**

Although conventional interventions for people at high risk of developing type 2 diabetes are usually conducted face-to-face, such interventions are burdensome for health care providers. We developed a lifestyle intervention program combining lifestyle coaching via a smartphone application augmented by intermittently scanned continuous glucose monitoring without burdening health care providers. Its effectiveness for glycemic control and body weight reduction in people at risk of type 2 diabetes was investigated.

**Materials and Methods:**

For this 12-week randomized unblinded trial with offline recruitment, participants with a hemoglobin A1c level of 5.6% to 6.4% or a fasting blood glucose of 110 to 125 mg/dL and body mass index (BMI) >23 kg/m^2^ but <40 kg/m^2^ were randomly assigned to the intervention group (App) and control group (C). The primary endpoint was the difference in time in range of blood glucose between 70 and 140 mg/dL (3.9-7.8 mmol/L) before and after the study period between the 2 groups.

**Results:**

Among 168 patients (mean age, 48.1 years; mean BMI, 26.6 kg/m^2^; and male, 80.4%), 82 and 86 were assigned to the App group and C group, respectively. After 12 weeks, time in range of blood glucose at 70 to 140 mg/dL significantly improved in the App group compared with the C group (−2.6 minutes/day vs +31.5 minutes/day, *P* = .03). Changes in time above range did not differ, whereas time below range (blood glucose <70 mg/dL; +23.5 minutes/day vs −8.9 minutes/day, *P* = .02) improved in the App group. BMI (−0.26 vs −0.59, *P* = .017) was reduced in the App group compared with the C group.

**Conclusion:**

Intervention with a smartphone app and intermittently scanned continuous glucose monitoring increased glycemic control accompanied by decreased carbohydrate intake and weight loss. Further trials are needed to confirm whether these interventions can reduce incident type 2 diabetes.

Diabetes mellitus is a risk factor for cardiovascular disease and has been associated with 2- to 4-fold higher mortality than in those without diabetes (1). Because diabetes adversely affects quality of life, delaying or preventing its onset is urgent and relevant in clinical settings. Prediabetes defined by hemoglobin A1c (HbA1c) levels of 5.7% to 6.4% present a high risk for type 2 diabetes (2). Whereas severe obesity was shown to increase the risk of type 2 diabetes in Western countries (3), a slightly increased body mass index (BMI; >23 kg/m^2^) was a risk factor for type 2 diabetes in Asians, including Japanese (4).

Weight reduction through lifestyle modifications (5-10), including diet and physical activity, is important in preventing type 2 diabetes. Effectiveness of such modifications in suppressing worsening of glycemic control has been established in persons at high risk of type 2 diabetes (11, 12). Conventional interventions are usually conducted by health care providers face-to-face, which is not only a burden on health care providers but varies in quality.

Lifestyle interventions to prevent type 2 diabetes based on Internet of Things devices, such as smartphone apps (13), are expected to reduce the burden on health care providers as well as ensure quality of educational programs. Previously, a 1.2% reduction in HbA1c was achieved using a smartphone app with which people with type 2 diabetes recorded daily activities and modified their lifestyle through responses to lifestyle intervention messages (14). Also, a review of 21 studies found that smartphone app-based interventions improved HbA1c in people with type 2 diabetes by 0.44% (15). Similarly, lifestyle interventions based on Internet of Things devices were effective for weight management in those with type 2 diabetes, (16). However, only a few randomized controlled trials (17, 18) have evaluated the effectiveness of interventions centered on smartphone applications for blood glucose control and weight reduction in people without type 2 diabetes but who are at high risk of its development.

Intermittently scanned continuous glucose monitoring (isCGM), which is minimally invasive, is used to manage diabetes. Monitoring blood glucose with isCGM not only provides health care providers with information but patients with appropriate feedback on diet, exercise, and medications, which would be expected to improve blood glucose control (19) and lead to weight reduction (20). Some reports on isCGM as a lifestyle intervention in people at high risk of type 2 diabetes showed adherence and satisfaction (21-24, 25). However, effects of its use on glycemic fluctuations and body weight (BW) remain unknown.

We developed a lifestyle intervention program that combines lifestyle coaching via a smartphone application with isCGM, which does not burden health care providers, and investigated its effectiveness for blood glucose control and BW reduction in people at high risk of type 2 diabetes.

## Methods

### Study Design

This is a prospective, randomized, unblinded 1-to-1 allocation trial. Those at high risk of type 2 diabetes, such as those with an HbA1c >5.6% and BMI >23, were allocated to either the intervention group, which received lifestyle coaching via a smartphone application and a variety of tests and examinations, or the control group, which underwent the tests and examinations only but no interventions.

### Approval and Ethical Considerations

This study was registered in the University Hospital Medical Information Network (UMIN000046400) and approved by the Ethics Committee of Niigata University. It was conducted in accordance with the Declaration of Helsinki (2013 revision) and the Ethical Guidelines for Medical and Biological Research Involving Human Subjects (2021). All participants provided written informed consent. Enrollment was from December 2021 to February 2022, and the study period was from December 2021 to May 2022.

### Study Participants, Inclusion Criteria, and Randomization

The study was conducted among employees of Sompo Japan Himawari Life Insurance Inc. and Sompo Japan Insurance Inc. and their affiliated companies. Research personnel from Niigata University recruited study participants online at each company through a videoconferencing system. The objectives of this research were explained including the information that the control group would not receive any intervention but only examinations. Those who agreed to participate were screened, after which individuals who met the inclusion criteria and gave written informed consent were enrolled as study participants.

Inclusion criteria were HbA1c 5.6% to 6.4% or fasting blood glucose 110 to 125 mg/dL, BMI >23 kg/m^2^ but <40 kg/m^2^, age 20 to 80 years, and possession of the smartphone app, Health2Sync app., and the ability to use it. Initial inclusion criteria had been HbA1c 5.7% to 6.4% and BMI ≥25 kg/m^2^, but because of insufficient enrollment, criteria were changed as described previously. Persons with diabetes mellitus, undergoing cancer treatment, and/or taking corticosteroids were excluded because they were deemed inappropriate by investigators.

Study participants were randomly assigned (1:1) to either the smartphone app and isCGM intervention group or the control group on a 1:1 basis according to sex, age (≤45 years or >45 years), and preassignment HbA1c (≥6.0% or <6.0%) using the minimization method with a web-based electronic data capture system (UHCT ACReSS) provided by a third party (University Hospital Clinical Trial Alliance, Tokyo, Japan). Neither the researchers nor the participants were blinded.

### Sample Size Design

The degree of improvements in time in range (TIR) after interventions with isCGM and smartphone apps in people at high risk for type 2 diabetes have not been well reported. A study of 10 days of carbohydrate restriction among those with prediabetes showed a 2.6% (SD 1.6%) improvement in TIR of blood glucose 70 to 140 mg/dL (22). In the current study, because carbohydrate restriction was not directly supervised, the average improvement would be small. On the other hand, after a follow-up of 3 months, values in some participants could be expected to either improve or worsen to a great extent, resulting in large variability. As a result, we estimated that TIR of blood glucose 70 to 140 mg/dL would improve by 30 minutes (2.1%) in the intervention group, whereas there would be no change in the control group. Standard deviations were estimated to be 5.5% in each group, with significance set at 0.05 (1-sided). Assuming a power of 80% and a drop-out rate of 15%, 86 patients per group were required. Therefore, the number of patients enrolled was set at 200, with 100 in each group.

### Support Provided by Smartphone App to the Intervention Group

Neither the intervention group nor the control group received input by a health care provider. As explained, the control group received no intervention. The intervention group used the smartphone app “Health2Sync” (Health2Sync Co., Taipei, Taiwan) and isCGM with “FreeStyle Libre Link” (Abbott Diabetes Care, Witney, UK), which is a lifestyle improvement program under development as an enhanced version of the Health2Sync and is not yet available to the public in Japan. It was believed that this instrument could provide information that might otherwise be provided by medical professionals.

**Figure 1.**
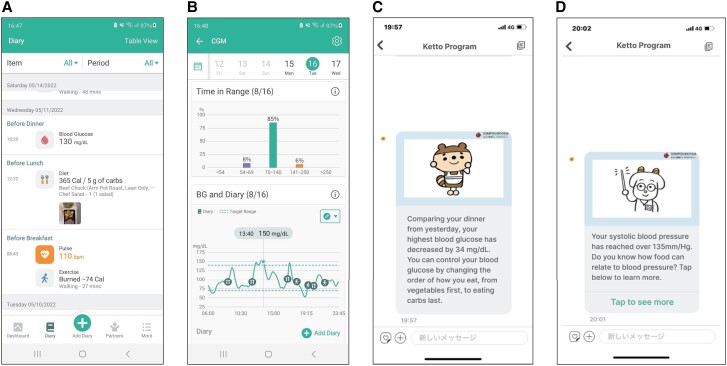
Screen shots of the Health2Sync app. A, Records of daily activities. B, Continuous glucose monitoring and daily activities. C, Message based on continuous glucose monitoring data. D, Message based on blood pressure data.

The Health2Sync mobile app, with more than 1 million users globally, supports patients with chronic diseases such as diabetes (26). It fosters favorable behavioral changes by tracking such changes and helping persons gain insights into their habits using features such as food records, physical activity records (manually or through a fitness tracking device), and diabetes-related information such as medications, BW, and blood pressure (Fig. 1A). Participants can review their behavior to improve habits. This version of Health2Sync (version 2.8.3 for both iOS and Android used in this study) also delivers messages based on a preset lifestyle intervention, providing information and advice on diet and exercise to prevent onset of type 2 diabetes. Personalized messages are delivered based on data input, such as continuous glucose monitoring data imported automatically through Freestyle Libre Link and food records, physical activity records, BW, and blood pressure input throughout Health2sync. Some messages have in-app links to more detailed information (Fig. 1C-D).

The app can be connected to FreeStyle Libre Link, which displays continuous blood glucose data obtained from isCGM in synchrony with meals and physical activity (Fig. 1B) to show relationships among blood glucose fluctuations and the timing of diet and exercise. Also, blood glucose fluctuations such as time in range and difference in values from the previous day are clearly displayed on Health2Sync. FreeStyle Libre is a widely used isCGM device that can be used either with a reader that also acts as a self-monitoring blood glucose device or with a smartphone application. Several versions of FreeStyle Libre (Freestyle Libre 1, 2, and 3) are available in some regions, but in Japan, only FreeStyle Libre 1 was available at the time of this study. We used FreeStyle Libre 1 with the FreeStyle Link smartphone application.

### Study Protocol and Data Collection

**Figure 2.**
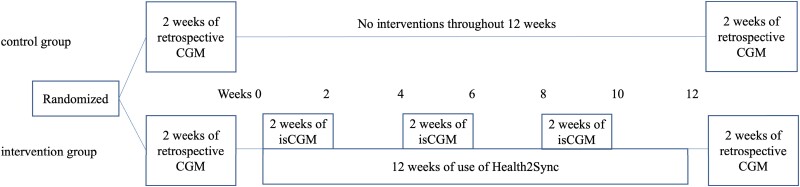
Use of smartphone applications, isCGM, and retrospective CGM.

Baseline data were obtained for both the intervention group and control group for the 2 weeks before a 12-week period during which the intervention group received the 12-week Health2Sync and isCGM intervention and the control group received no lifestyle modification information and were prohibited from using health care-related smartphone applications. Table 1 shows the baseline characteristics and results of physical examinations, blood tests, blood glucose measurements with retrospective CGM, and questionnaires obtained 2 weeks before the study period. Freestyle Libre and other instruments for measurements, blood test kits, questionnaires, and blood pressure monitors were mailed to the participants' homes by the researchers. In both groups, retrospective CGM using FreeStyle Libre Pro was performed to obtain data on blood glucose variability for 2 weeks before the 12-week study period, during which the intervention group used Health2Sync and the control group received no intervention through these instruments. The results of retrospective CGM were blinded to the participants. Blood testing was performed using a kit (27) (Leisure, Inc., Tokyo, Japan) that required a fingertip blood sample collected at home by the participant and that was send to the laboratory. Energy intake assessed by the Brief Diet History Questionnaire (28) and physical activity assessed by the Japanese version of the International Physical Activity Questionnaire short form (29, 30) were collected before and after the 12-week study period in both groups. The use of any smartphone application by the intervention group that would contribute to improving lifestyle habits other than the Health2Sync app and FreeStyle Libre Link was prohibited during the study period. Details of the protocol are provided in Fig. 2.

**Table 1.** Baseline characteristics of study participants

		Control		Intervention
Sex (M/F)	82	66/16	86	69/17
Age (y)	82	47.38 ± 7.49	86	49.19 ± 8.75
Body weight (kg)	82	76.34 ± 11.45	86	76.33 ± 13.21
Body weight index (kg/m^2^)	82	26.40 ± 3.30	86	26.57 ± 3.77
Waist circumference (cm)	63	92.2 ± 8.96	68	92.2 ± 9.14
Systolic blood pressure (mm Hg)	64	124.8 ± 12.54	68	126.01 ± 15.8
Diastolic blood pressure (mm Hg)	64	81.42 ± 10.72	67	83.73 ± 11.59
HbA1c (%)	74	5.81 ± 0.34	83	5.76 ± 0.31
Triglycerides (mg/dL)	74	132.5 (95-179)	83	127 (87-181)
Total cholesterol (mg/dL) ‡	74	206.09 ± 27.62	83	200.35 ± 27.01
HDL-C (mg/dL)	74	63.69 ± 14.62	83	63.89 ± 14.2
LDL-C (mg/dL)	74	129.57 ± 28	83	125.22 ± 26.75
Protein (g/day)	79	62.28 ± 22.07	81	66.84 ± 24.83
Fat (g/day)	79	53.09 ± 18.15	81	54.45 ± 19.72
Carbohydrate (g/day)	79	197.98 ± 66.04	81	227.38 ± 82.37
Physical activity (METs h/week)	79	13.8 (6.6-23.3)	81	14.6 (6.6-24.2)
Mean glucose (mg/dL)	77	104.01 ± 10.06	76	105.19 ± 12.07
SD (mg/dL)	77	22.12 ± 5.78	76	22.7 ± 6.55
CV (%)	77	21.18 ± 4.77	76	21.42 ± 4.6
> 180 mg/dL (min/day)	77	20.6 ± 33.6	76	27.4 ± 56.7
> 140 mg/dL (min/day)	77	120.5 ± 111.4	76	129.1 ± 138
70-180 mg/dL (min/day)	77	1380.8 ± 75.3	76	1377.3 ± 70
70-140 mg/dL (min/day)	77	1280.9 ± 120.2	76	1275.6 ± 134.9
101-140 mg/dL (min/day)	77	543.1 ± 183.1	76	563.2 ± 181.5
70-100 mg/dL (min/day)	77	737.8 ± 232.6	76	712.4 ± 245.3
54-140 mg/dL (min/day)	77	1311.3 ± 109.5	76	1305.0 ± 136
< 70 mg/dL (min/day)	77	38.6 ± 71	76	35.3 ± 53.9
54-69 mg/dL (min/day)	77	30.5 ± 53.1	76	29.4 ± 46.2
< 54 mg/dL (min/day)	77	8.2 ± 26.2	76	5.8 ± 17
CONGA	77	84.55 ± 15.58	76	86.69 ± 13.66
MAGE	77	56.07 ± 19.44	75	56.55 ± 16.08

Data are presented as mean ± standard deviation, median (25%, 75% percentile).

Abbreviations: CONGA, continuous overlapping net glycemic action; CV, coefficient of variation; F, female; HbA1c, hemoglobin A1c; HDL-C, high-density lipoprotein cholesterol; LDL-C, low-density lipoprotein cholesterol; M, male; MAGE, mean amplitude of glucose excursion; METs, metabolic equivalents.

During the intervention period, data such as on BW, waist circumference, and systolic and diastolic (DBP) blood pressure were measured at home by intervention group participants at weeks 0, 2, 4, 6, 8, 10, and 12 during the 12-week intervention. These participants were asked to wear the FreeStyle Libre on days 1 through 14, 29 through 42, and 57 through 70 to monitor glucose fluctuations. The Health2Sync app was used throughout the 12-week study period to record lifestyle-related parameters such as weight, diet, and physical activity and to receive messages to encourage improvements. The intervention was repeated in 3 sets of 2 weeks (6 weeks) using Freestyle Libre Link and Health2Sync together and in 3 sets of 2 weeks (6 weeks) using only Health2Sync, for a total of 12 weeks. During the 2-week period of combined use of Freestyle Libre Link and Health2Sync, the participants learned about the relationship between changes in blood glucose and lifestyle from CGM-related messages from the Health2Sync app. In the 2-week periods when only Health2Sync was used, participants worked on applying lifestyle modifications based on findings during FreeStyle Libre use. Then after the 12-week study period, similar to the before the study period, physical examinations, blood tests, blood glucose measurements by retrospective CGM, and a questionnaire survey were conducted.

### Study Outcomes

The primary endpoint was the difference in TIR of blood glucose 70 to 140 mg/dL (3.9-7.8 mmol/L) obtained by retrospective CGM before and after the intervention between the control and intervention groups. No baseline corrections or model adjustments were made.

The secondary endpoints were the difference between the start and finish of the study period between the 2 study groups in systolic blood pressure, DBP, BW, BMI, waist circumference, HbA1c, TIR of blood glucose 70 to 180 mg/dL, time above range (TAR) of blood glucose >140 mg/dL, TAR of blood glucose >180 mg/dL, time below range (TBR) of blood glucose <70 mg/dL, TBR of blood glucose <54 mg/dL, SD, coefficient of variation (CV), mean amplitude of glucose excursion (MAGE), continuous overlapping net glycemic action at 2 hours (CONGA 2 hours), nutrient intake, and physical activity.

Health2Sync app usage was evaluated by the number of days the app was opened between days 1 and 14, 15 and 28, 29 and 42, 43 and 56, 57 and 70, and 71 and 84 and the number of food logs and the number of physical activity logs. FreeStyle Libre Link usage was assessed by the frequency of blood glucose levels recorded during days 1 and 14, 29 and 42, and 57 and 70.

For the exploratory analysis, a stratified analysis was performed according to baseline TIR of blood glucose 70 to 100 mg/dL or 101 to 140 mg/dL and baseline HbA1c of ≥5.8% or <5.8%. Regression analysis of factors contributing to changes in glycemic variability indices and BW in the control group was also performed. Furthermore, the percentage of participants in each group who achieved a weight reduction of ≥2 kg was examined.

### Safety Analysis

Times that blood glucose was <70 mg/dL or <54 mg/dL were recorded and indicated hypoglycemia or severe hypoglycemia, respectively. Symptoms related to sensor insertion and wear were recorded.

### Statistical Analysis

Analyses of the primary and secondary endpoints were primarily performed on the full analysis set (FAS), which included all participants assigned to a study group. Analysis of the per protocol set (PPS) was performed to confirm the stability of the results for the primary and secondary endpoints. Two groups were compared by the Student *t*-test and Wilcoxon rank sum test according to distribution. The χ^2^ test was used to determine the percentage of those achieving a weight reduction of ≥2 kg. Significance level was 0.05 (1-sided). Also, we compared the amount of change between the 2 groups for each endpoint using an analysis of covariance with each baseline characteristic as an adjustment variable.

If there were no measured values, data were considered missing, and no imputation was performed. All statistical analyses were performed using SPSS (version 27.0, Chicago, IL, USA) by an investigator who is a statistics specialist and was not involved in recruiting participants.

## Results

### Data Available for Analysis in the Intervention Group and Control Group

Of the 236 people who expressed willingness to participate, written informed consent was obtained from 179. Excluded were 57 individuals who did not meet the eligibility criteria. Eighty-eight persons were assigned to the control group and 91 to the intervention group. The FAS analysis was performed on 168 participants with 82 in the intervention group and 86 in the control group, excluding 11 patients who did not undergo an examination or intervention after allocation (6 participants, control group; 5 participants, intervention group).

**Figure 3.**
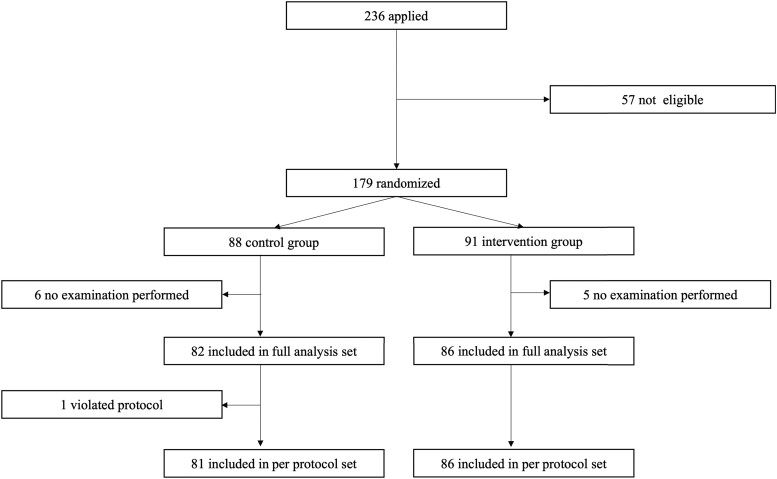
Flow chart of participants.

Of the remaining participants, 153 and 121 had blood glucose fluctuation data before and after the study period, respectively. As a result, the primary endpoint was analyzed in 116 patients who had blood glucose fluctuation data both before and after the study period. Secondary endpoints derived from evaluating changes before and after the intervention were determined. One control group participant was later deemed not to meet the inclusion criteria and was excluded from the PPS analysis, resulting in a PPS analysis of 167 participants. Safety analysis included 168 participants, the same as for the FAS. Details of the disposition of the participants are shown in Fig. 3.

### Results of Analysis

Baseline characteristics for the entire study population before the allocation were mean age 48.1 years, 80.4% male, mean BMI 26.6 kg/m^2^, and HbA1c 5.8% (Table 1). Characteristics of 116 patients included in the primary endpoint analysis were mean age 48.6 years, 75.9% male, mean BMI 26.2 kg/m^2^, and HbA1c 5.8% (Supplementary Table 1) (31), which were similar to those in the total participant population.

**Figure 4.**
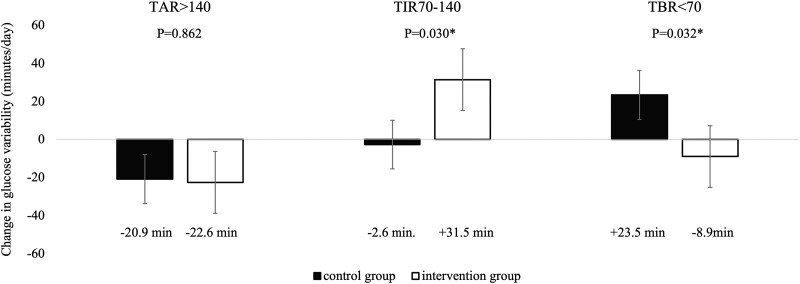
Changes in glucose variability between the control group and the intervention group. Error bars in the table indicate standard error.

**Figure 5.**
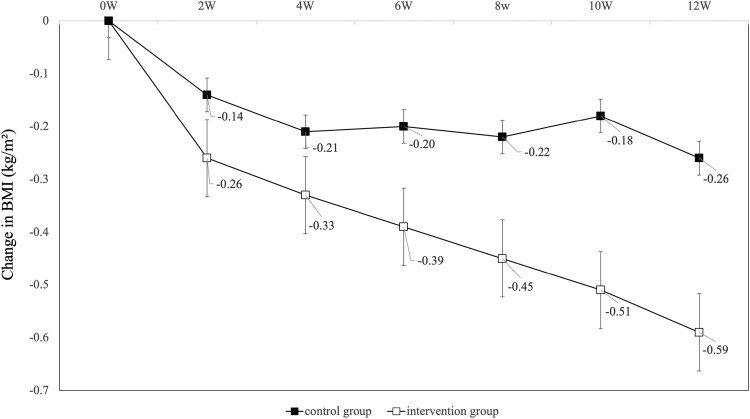
Changes in body mass index at weeks 0, 2, 4, 6, 8, 10, and 12. Patients with fasting biochemical data at each time point were included in the analysis. Error bars in the table indicate standard errors.

In the analysis of the primary endpoint, change in TIR of blood glucose 70 to 140 mg/dL before and after the intervention was significantly in the intervention group compared with the control group (control group; −2.7 minutes/day vs intervention group; +31.5 minutes/day, *P* = .030) (Fig. 4). The improvement in TIR of blood glucose 70 to 140 mg/dL in the intervention group was due to a 64.7-minute decrease in TIR of blood glucose 101 to 140 mg/dL and a 96.2-minute increase in TIR of blood glucose 70 to 100 mg/dL. Surprisingly, secondary endpoints such as changes in TAR of blood glucose >140 mg/dL did not differ between groups, whereas TBR of blood glucose <70 mg/dL improved in the intervention group (+23.5 minutes/day vs −8.9 minutes/day, *P* = .032). Regarding glycemic variability indices, CV improved (+0.62 vs −0.85, *P* = .029), but MAGE, CONGA 2 hours, and SD did not differ between groups. There were no significant differences in changes in HbA1c and mean glucose values between the 2 groups. In the intervention group, BMI (−0.26 vs −0.59, *P* = .017), waist circumference (−0.62 cm vs −1.51 cm, *P* = .042), and DBP (−0.5 mm Hg vs −4.0 mm Hg, *P* = .004) improved significantly. BMI decreased significantly in the intervention group from 2 weeks after baseline and continued to decrease over the 12-week intervention period (Fig. **5**). Weight reductions of ≥2 kg were noted in 22 (32.8%) participants in the intervention group and in 11 (15.9%) in the control group, with a significantly higher value in the intervention group (*P* = .028).

Carbohydrate intake (−4.4 kcal/day vs −22.7 kcal/day, *P* = .049) was significantly reduced in the intervention group compared with the control group. Conversely, the change in total energy intake did not differ between groups (−60.58 kcal/day vs −176.38 kcal/day, *P* = .156). Neither was there a between-group difference in the change in physical activity (+4.06 metabolic equivalents h/week vs −0.72 metabolic equivalents h/week, *P* = .188). Details of changes in variables from 0 to 12 weeks are shown in Table 2.

**Table 2.** Changes in each variable from week 0 to week 12

		Control		Intervention	*P*
Δ_Body weight (kg)	69	−0.77 ± 1.82	67	−1.7 ± 2.73	.021
Δ_Body weight index (kg/m^2^)	69	−0.26 ± 0.62	67	−0.59 ± 0.93	.017
Δ_Waist circumference (cm)	62	−0.62 ± 1.9	67	−1.51 ± 2.96	.042
Δ_Systolic blood pressure (mm Hg)	63	−2.43 ± 8.01	67	−3.81 ± 9.71	.381
Δ_Diastolic blood pressure (mm Hg)	63	−1.67 ± 5.87	67	−4.51 ± 6.95	.013
Δ_HbA1c (%)	63	−0.03 ± 0.31	65	−0.06 ± 0.2	.45
Δ_Protein (g/day)	71	−0.2 (−11-5.8)	71	−1.5 (−13.3-9.6)	.746
Δ_Fat (g/day)	71	−0.6 (−12-8)	71	−2.4 (−11.9-7.8)	.866
Δ_Carbohydrate (g/day)	71	−4.4 (−37.9-20.9)	71	−22.7 (−70-18.5)	.049
Δ_Physical activity (METs h/week)	72	0 (−4.9-8.3)	72	0 (−5.5-7.6)	.931
Δ_Mean glucose (mg/dL)	58	−3.89 ± 6.58	58	−2.66 ± 9.46	.419
Δ_SD (mg/dL)	58	−0.29 ± 2.91	58	−1.43 ± 4.94	.131
Δ_CV (%)	58	0.62 ± 3.04	58	−0.85 ± 4.03	.029
Δ_>180 mg/dL (min/day)	58	−5.5 ± 16.9	58	−8.4 ± 39.1	.786
Δ_>140 mg/dL (min/day)	58	−20.8 ± 50.6	58	−22.6 ± 84	.862
Δ_70-180 mg/dL (min/day)	58	−18.0 ± 73.1	58	17.3 ± 70	.007
Δ_70-140 mg/dL (min/day)	58	−2.7 ± 79.9	58	31.5 ± 87.7	.030
Δ_101-140 mg/dL (min/day)	58	−71.1 ± 162.8	58	−64.7 ± 194.3	.847
Δ_70-100 mg/dL (min/day)	58	68.5 ± 177.9	58	96.2 ± 222.7	.461
Δ_54-140 mg/dL (min/day)	58	14.6 ± 52.1	58	27.8 ± 83.1	.252
Δ_<70 mg/dL (min/day)	58	23.5 ± 76.5	58	−8.9 ± 66.5	.032
Δ_54-69 mg/dL (min/day)	58	17.3 ± 57.1	58	−3.7 ± 58.2	0.093
Δ_<54 mg/dL (min/day)	58	6.2 ± 31.1	58	−5.2 ± 19.7	.167
Δ_CONGA	58	−2.5 (−7.2-0.7)	58	−1.6 (−7.1-2.7)	.307
Δ_MAGE	56	−1.8 (−7.9-2.4)	58	−3.4 (−9.9-6.3)	.755

Data are presented as mean ± SD, median (25%, 75% percentile).

Abbreviations: CONGA, continuous overlapping net glycemic action; CV, coefficient of variation; HbA1c, hemoglobin A1c; MAGE, mean amplitude of glucose excursion; METs, metabolic equivalents.

In an analysis of covariance, in which the group differences in changes for each item were performed with a baseline　characteristic as the adjusted variable, TIR of blood glucose 70 to 140 (*P* = .046) and TBR of blood glucose <70 (*P* = .025) showed significant (*P* = .046) improvement, as did the prespecified main analysis. TIR of blood glucose 70 to 180 (*P* = .016) was also significantly improved in the intervention group. Other endpoints were similar to the main analysis. In the main analysis, dietary intake, physical activity, CONGA, and MAGE were analyzed based on the Wilcoxon rank sum test according to distribution, so the difference in *P* values between the main analysis and the additional analysis was greater than for the other items analyzed with the Student *t* test. The change in carbohydrates was significantly improved in the main analysis but not in this additional analysis. The change in CONGA did not differ significantly in the main analysis but the change was significant in this additional analysis (see detail in Supplementary Table 4) (31).

The number of intervention group participants with isCGM data for days 1 to 14, 29 to 42, and 57 to 70 were 72 (83.7%), 68 (79.1%), and 62 (72.1%), respectively. For those with isCGM data, isCGM use for those days was 1138 minutes/day (79.0%), 1093 minutes/day (75.9%), and 1045 minutes/day (72.6%), respectively. The number of participants with Health2Sync app data on days 1 to 14, 15 to 28, 29 to 42, 42 to 56, 57 to 70, and 71 to 84 was 72 (83.7%), 65 (75.6%), 69 (80.2%), 58 (67.4%), 64 (74.4%), and 57 (66.3%), respectively. For those with Health2Sync app data, the number of days that the smartphone application was opened during days 1 to 14, 15 to 28, 29 to 42, 42 to 56, 57 to 70, and 71 to 84 was 11.3, 8.6, 9.4, 7.5, 8.4, and 6.4 days, respectively. Also, the number of days/2 weeks that participants entered meal records was 7.8, 4.4, 5.9, 3.7, 5.0, and 2.7 days, respectively, within the periods for days 1 to 14, 15 to 28, 29 to 42, 42 to 56, 57 to 70, and 71 to 84. For similar periods, physical activity records were entered during 3.1, 2.3, 2.5, 2.4, 2.1, and 1.4 days, respectively (see details in Table 3). The FAS for the 168 participants and the PPS analysis of 167 participants, excluding that 1 participant mentioned previously, showed results similar to those of the FAS analysis. Details are shown in Supplementary Tables 2 and 3 (31).

**Table 3.** Usage of isCGM and smartphone app in the intervention group

	Days 1-14	Days 15-28	Days 29-42	Days 43-56	Day 57-70	Days 71-84
Number of participants with Freestyle Libre	72		68		62	
Time with isCGM data (%)	79.0 ± 21.0		75.9 ± 21.2		72.6 ± 23.0	
Number of participants with Health2Sync app data	72	65	69	58	64	57
Number of days						
Health2Sync was open (times/2 weeks)	11.3 ± 3.9	8.6 ± 4.8	9.4 ± 4.7	7.5 ± 4.8	8.4 ± 5.0	6.4 ± 4.7
With food records (times/2 weeks)	7.8 ± 5.4	4.4 ± 5.5	5.6 ± 6.2	3.7 ± 5.1	5.0 ± 5.7	2.7 ± 4.2
With physical activity records (times/2 weeks)	3.1 ± 3.8	2.3 ± 3.6	2.5 ± 3.9	2.4 ± 3.7	2.1 ± 3.4	1.4 ± 2.4
With blood pressure records (times/2 weeks)	3.1 ± 5.0	2.3 ± 4.2	2.2 ± 4.5	2.0 ± 3.9	2.1 ± 4.0	1.4 ± 3.3
With body weight records (times/2 weeks)	2.7 ± 4.2	2.2 ± 4.0	2.2 ± 4.3	1.8 ± 3.5	2.0 ± 4.0	1.6 ± 3.6

Data are presented as mean ± SD.

Abbreviation: isCGM, intermittently scanned continuous glucose monitoring.

### Exploratory Analysis

The results of the analysis according to baseline HbA1c values are shown in Supplementary Tables 5 and 6 (31). Baseline TAR of blood glucose >140 tended to be longer in the group with HbA1c ≥5.8% compared with the group with HbA1c <5.8%, whereas the TBR of blood glucose <70 did not change according to HbA1c values. There was also a tendency for a greater reduction in TBR of blood glucose <70 and improvement in CV and other glycemic variables in the group with HbA1c ≥5.8%.

We also examined whether lifestyle changes and the usage of isCGM and smartphone apps contributed to the improvement in glycemic variability and BMI. Changes in diet and physical activity did not contribute to the improvement of TIR of blood glucose 70 to 140 mg/dL, but decreases in carbohydrate and fat intakes and increases in protein intake did contribute to weight loss (Supplementary Table 7) (31). Use of isCGM and the smartphone app use did not contribute to improvements in glucose variability or BMI (Supplementary Table 8) (31).

### Safety Analysis

Hypoglycemia with blood glucose values <70 mg/dL was recorded for 38.6 minutes/day (2.68%) in the control group and 35.3 minutes/day (2.45%) in the intervention group in the 2 weeks before the intervention and for 53.9 minutes/day (3.74%) in the control group and 31.1 minutes/day (2.16%) in the intervention group after the end of the 12-week intervention.

No severe hypoglycemia occurred that required medical care. Before the intervention, severe hypoglycemia with blood glucose values <54 mg/dL took place for 8.2 minutes/day (0.57%) in the control group and 5.8 minutes/day (0.40%) in the intervention group. After the intervention, this condition occurred for 8.9 minutes/day (0.62%) in the control group and 3.6 minutes/day (0.24%) in the intervention group.

Eight adverse events in 5 participants associated with FGM puncture occurred in the control group (4 pain, 2 bleeding, 2 itching) and 13 events in 11 participants in the intervention group (9 pain, 2 bleeding, 2 itching), with no severe adverse events leading to discontinuation of the study.

## Discussion

This is the first study of the efficacy of a lifestyle intervention using isCGM and a smartphone application that does not require interventions by health care providers and maintains a consistent level of quality. The intervention group showed a moderate but significant increase in the primary endpoint of TIR of blood glucose 70 to 140 mg/dL, accompanied by weight reduction and a decrease in carbohydrate intake. Also, improvements in blood glucose fluctuations were noted.

Previously, type 2 diabetes prevention has focused on in-person interventions from health care providers, such as the Diabetes Prevention Program (11). However, expectations for non-face-to-face interventions using new information and communication technology such as the internet, telephone, and smart phone applications increased because they reduce the burden on both patients and health care providers and help to maintain the quality of intervention programs. Lifestyle interventions using smartphone apps can be categorized into 2 types: those involving a health care provider via a smartphone app and those automatically performed by an algorithm in the smartphone app without a health care provider. A systematic review of smartphone apps for type 2 diabetes and hypertension patients (15) indicated that some participation by a health care provider was more effective than the smartphone app alone.

Interventions with some medical provider's input are classified into 2 categories: conventional type 2 diabetes prevention programs conducted via a smartphone application (13) and lifestyle coaching taking full advantages of the features of the smartphone application (17, 22). Although both have been reported to be effective in people at high risk for type 2 diabetes, involvement of health care providers remains in any case. There are few reports on the effectiveness of a smartphone application alone without involving health care providers in improving glycemic variability and weight reduction in people at high risk for type 2 diabetes.

Our program provided a highly personalized intervention in place of a health care provider's intervention by combining 2 kinds of messages: regularly delivered type 2 diabetes prevention guidance and messages personalized by an algorithm based on dietary, physical activity, and blood glucose monitoring records. As a result, blood glucose fluctuations, BW, and carbohydrate intake were improved, which revealed the effectiveness of the smartphone application in combination with appropriately personalized and automated interventions, such as the isCGM, in those at high risk of type 2 diabetes.

In this study, the primary endpoint, TIR of blood glucose 70 to 140 mg/dL, was prolonged by 33 minutes/day mainly because of the increase of TIR of blood glucose 70 to 100 mg/dL in the intervention group. We focused on postprandial hyperglycemia and expected a reduced duration of hyperglycemia with interventions designed to prevent postprandial hyperglycemia. However, contrary to our expectations, there was no difference in the change in TAR of blood glucose >140 mg/dL and a decrease in TBR of blood glucose <70 mg/dL. We cannot clearly explain why the TBR of blood glucose <70 mg/dL increased in a population not using hypoglycemic medications. It is possible that we overestimated the TBR for blood glucose <70 mg/dL because continuous glucose monitoring systems are generally designed for diabetes patients and are inherently not well validated in nondiabetes patients. In the latter case, false low blood glucose values are often experienced. However, clearly there was a decrease in hypoglycemic tendencies in the intervention group as well as an increase in the appropriate range of blood glucose compared with the control group. We speculate that there was minor improvement in postprandial hyperglycemia with blood glucose not exceeding 140 mg/dL, with a subsequent reduction in blood glucose levels.

Even those without type 2 diabetes and not using hypoglycemic agents have been reported to develop unpleasant symptoms such as postprandial syndrome associated with postprandial blood glucose fluctuations. Postprandial syndrome can occur in the absence of hyperglycemia and subsequent overt hypoglycemia (32, 33) and is associated with high-carbohydrate diets and hyperinsulinemia (34). In fact, the mean HbA1c of the study population was 5.8%, whereas participants with HbA1c of 5.6% to 5.8% were reported to have excessive insulin secretion compared with those with HbA1c <5.5% and HbA1c >5.9% (35). Also, in the analysis stratified by HbA1c values, there was a trend toward greater improvement in the times during which hyperglycemia and hypoglycemia were present and glycemic variability indices in the group with higher HbA1c, suggesting that glycemic variability may have been better controlled.

As initially intended, use of our smartphone application designed to prevent elevated postprandial hyperglycemia reduced carbohydrate intake and postprandial blood glucose variability. In addition, although factors that cause postprandial syndrome, such as postprandial hyperinsulinemia, were not measured, these conditions may have improved. Also possible is that the improvement in postprandial syndrome was associated with an improvement in quality of life. However, the duration of hyperglycemia did not improve and mean blood glucose levels were not reduced. Further research is needed to determine whether these small changes in blood glucose levels contribute to the prevention of type 2 diabetes, independent of changes in BW and other factors.

Weight loss through diet and physical activity (5-8, 36, 37) is important in preventing type 2 diabetes.

In this study, we focused on postprandial hyperglycemia, which is common in those with impaired glucose tolerance, and used a dietary message that focused on controlling blood glucose fluctuations, especially avoiding excessive carbohydrate intake. In those with type 2 diabetes, a continuous glucose monitoring intervention was reported to improve dietary habits (38, 39). Although the current study was of those without type 2 diabetes, the use of isCGM in combination with messages allowed a more effective dietary intervention. As expected, our program resulted in reduced carbohydrate intake, which is associated with postprandial hyperglycemia and other glycemic variations, and improved glycemic variability, as the primary endpoint of our study.

In terms of the amount of physical activity, we found no improvement in the intervention group, despite the extensive use of messages encouraging increased physical activity, including exercise videos. The questionnaire we used to assess physical activity in this study underestimated short bouts of physical activity, such as <10 minutes, as a limitation of the questioning method. Further research, such as the use of a physical activity monitor to assess physical activity, is needed.

Weight reduction was significant from 2 weeks after the start of the intervention, and the effect continued until after the 12-week intervention with the difference between the intervention and control groups increasing to 1 kg. Furthermore, weight reductions of ≥2 kg, which has been reported to be effective in preventing diabetes in Japanese (8-10), were significantly more frequent in the intervention group.

The effect of isCGM on BW can vary in those with type 2 diabetes, depending on their insulin regimen (40). This is possibly because of the increased use of insulin to control blood glucose, resulting from recognizing high blood glucose levels through isCGM. However, the use of basal insulin alone or oral antihyperglycemic agents often leads to weight loss (41, 42). Therefore, when study participants are not on insulin therapy, as in this study, weight reduction is likely to be achieved by improving lifestyle habits, particularly dietary intake. The smartphone application sent messages about improving dietary and exercise habits intended to reduce BW. In fact, the decrease in carbohydrate intake contributed to weight reduction. Although total energy intake or physical activity did not improve, the reduction in carbohydrate intake and subsequent reduction in blood glucose fluctuations, as well as the effect of isCGM, may have resulted in weight reduction.

This study has some limitations. First, to obtain sufficient statistical power for this study, we estimated that a total of 200 people with 100 in each group would need to be enrolled and 172 individuals would need to be analyzed. However, enrollment was limited to 179 persons, and data for the primary endpoint could not be obtained for 30 individuals in the control group and 33 individuals in the intervention group, resulting in only 116 persons analyzed for the primary endpoint. In addition, no previous studies have discussed the estimation of sample sizes, and it is possible that a reasonable sample size calculation could not be made. Although the primary endpoint was significantly improved, its interpretation needs to be noted. Second, this study was initially designed for persons with an HbA1c of 5.7% to 6.4% and BMI of ≥25 kg/m^2^, but these parameters were not favorable to enrollment, so we revised the inclusion criteria of an HbA1c to 5.6% to 6.4% and BMI of ≥23 kg/m Because these revised criteria certainly indicate a risk for type 2 diabetes (4, 43), at least in Japanese, this change does not reduce the value of our study. However, a more thorough consideration in planning the study would have been desirable. Third, the frequency of smartphone application use decreased over time, and only approximately 70% of participants completed all interventions and examinations. Although it has been reported that the use of smartphone applications and isCGM declines over time (13, 17, 18, 21), our decline rate was slightly higher than previous studies. This might be because this intervention study did not involve direct communication between health care providers and participants, but rather through mailed devices and a smartphone app program. Although we did not survey smartphone app satisfaction or dissatisfaction, future efforts should be made to increase compliance. Fourth, the duration of hyperglycemia did not improve. This may be due to the short duration of hyperglycemia in our participants who did not have type 2 diabetes. Although we applied the commonly used TIR of blood glucose 70 to 140 mg/dL, it is not yet clear to what extent a reduction in TIR contributes to the prevention of type 2 diabetes. More appropriate glycemic variability endpoints need to be investigated in the high-risk group for type 2 diabetes. Fifth, in this study, energy intake and physical activity were measured using questionnaires. Although they were sufficiently validated (28, 29, 30), their results may be influenced by various biases, such as recall bias. Further studies with objective methods, such as precise dietary records and physical activity meters, are needed to evaluate the effects of our intervention on lifestyle changes. Sixth, the majority of participants, 80%, were male. In addition, they were employees of Japanese life insurance companies. Therefore, extrapolation to the general population, including women, as well as to non-Japanese, must be questioned.

## Conclusion

A lifestyle intervention using isCGM and a smartphone app improved glycemic fluctuations and increased weight loss in people at high risk of type 2 diabetes. Further trials are needed to confirm whether these interventions can reduce incident type 2 diabetes in the future.

## Date Availability

The datasets that were analyzed during the current study are not available generally because of the data confidentiality policy of the Ethics Committee but may be obtained from the corresponding author upon a reasonable request and with the approval of the Ethics Committee.

## Clinical Trials Information

This study was registered in the University Hospital Medical Information Network (UMIN000046400).

## Abbreviations

BMI: body mass index
BW: body weight
CV: coefficient of variation
CONGA: continuous overlapping net glycemic action
DBP: diastolic blood pressure
FAS: full analysis set
HbA1c: hemoglobin A1c
isCGM: intermittently scanned continuous glucose monitoring
MAGE: mean amplitude of glucose excursion
PPS: per protocol set
TAR: time above range
TBR: time below range
TIR: time in range
